# The Edible Insect *Gryllus bimaculatus* Protects against Gut-Derived Inflammatory Responses and Liver Damage in Mice after Acute Alcohol Exposure

**DOI:** 10.3390/nu11040857

**Published:** 2019-04-16

**Authors:** Bo Byeol Hwang, Moon Han Chang, Jin Hyup Lee, Wan Heo, Jae Kyeom Kim, Jeong Hoon Pan, Young Jun Kim, Jun Ho Kim

**Affiliations:** 1 Department of Food and Biotechnology, Korea University, Sejong 30019, Korea; poi987@korea.ac.kr (B.B.H.); def92@korea.ac.kr (M.H.C.); jinhyuplee@korea.ac.kr (J.H.L.); 01062033526@korea.ac.kr (W.H.); 2 School of Human Environmental Sciences, University of Arkansas, Fayetteville, AR 72701, USA; jkk003@uark.edu (J.K.K.); jhpan@uark.edu (J.H.P.); 3 Department of Food Science and Biotechnology, Andong National University, Andong 36729, Korea

**Keywords:** *Gryllus bimaculatus*, alcoholic liver diseases, reactive oxygen species, intestinal permeability

## Abstract

Accumulation of reactive oxygen species (ROS) in response to excess alcohol exposure is a major cause of gut barrier disruption and lipopolysaccharide (LPS)-induced hepatic inflammation, as well as liver steatosis and apoptosis. This study was designed to investigate protective effects of the cricket *Gryllus bimaculatus*, an edible insect recognized by the Korea Food and Drug Administration, against acute alcoholic liver damage in mice. Administration of *G. bimaculatus* extracts (GBE) attenuated alcohol-induced steatosis and apoptotic responses in the liver and intestinal permeability to bacterial endotoxin. These protective effects were associated with suppression of ROS-mediated oxidative stress in both the liver and small intestine. Furthermore, in vivo and in vitro studies revealed that GBE inhibits LPS-induced Kupffer cell activation and subsequent inflammatory signaling. Importantly, the protective effects of GBE were more potent than those of silymarin, a known therapeutic agent for alcoholic liver diseases.

## 1. Introduction

The benefits of edible insects as an alternative animal protein source have recently drawn significant attention. Edible insects have great potential as highly nutritious, environmentally friendly, and economically feasible alternatives for the food industry in the future [1]. Insects as food are more sustainable compared with farm animals, in particular considering land use and global warming potential (sum of CO_2_, CH_4_, and NO_2_ emissions) [2]. In addition, the global market size of edible insects has been growing in and around South East Asia, showing that export and import of insects for food plays a strong economic role [3]. With increasing attention on edible insects as a food resource, there have been efforts to investigate their functional and pharmacological potential. Among various edible insects, the cricket *Gryllus bimaculatus* has a long history of traditional use in oriental medicine. It has been shown that it possesses anti-obesity, anti-aging, immunomodulatory, and hepato-protective properties [4,5,6,7], although underlying mechanisms are poorly understood. Recently, Ahn et al. have reported that glycosaminoglycan derived from *G. bimaculatus* has significant anti-inflammatory and anti-obesity effects in rodent models [8,9]. These findings provide important information for the use of *G. bimaculatus* as nutraceutical and/or pharmaceutical agent.

Alcoholic liver disease (ALD) is becoming increasingly severe worldwide, with the highest prevalence (around 12%) in Europe and the United States [10]. ALD is a multifaceted disorder that is characterized by a complex spectrum of hepatic steatosis, hepatitis, cirrhosis and/or hepatocellular carcinoma [11]. Multiple lines of evidence have suggested potential pathogenic mechanisms associated with ALD: key mechanisms identified include oxidative stress and inflammatory responses in the gut–liver axis, leading to the development and progression of ALD [12,13]. Reactive oxygen species (ROS) generated during ethanol (EtOH) metabolism can directly affect the transcriptional network that regulates gut permeability, hepatic lipogenesis, and apoptosis during liver injury [14], although there is limited information regarding the key source of ROS found in the gut during EtOH metabolism. In addition, ROS-induced oxidative stress promotes the release of pro-inflammatory cytokines that contribute to a tightly interrelated network between hepatocytes and macrophages in ALD [12].

Since ROS plays critical roles in the deterioration of ALD, various dietary antioxidants have been investigated for their potential use as adjuvants in the prevention and/or treatment of ALD [15]. The majority of animal studies have reported that antioxidants supplementation protects the liver from alcohol-induced injury by modulating cellular antioxidant system and inflammatory responses, as well as simply scavenging ROS [15,16] Based on the antioxidant capacity of *G. bimaculatus*, the aim of this study was to determine molecular mechanisms involved in the protection of *G. bimaculatus* extracts against ALD in mice with acute alcohol treatment. Because females are at a greater risk of ALD compared to males [17,18], here we used a female mouse model. With growing interest in the benefit of edible insects as future food resources, this study may provide important scientific clues for the utilization of *G. bimaculatus*.

## 2. Materials and Methods

### 2.1. Preparation of Edible Insect Extracts

Dried and ground *G. bimaculatus* was extracted with 10 volumes of distilled water at 60 °C or 70% EtOH at room temperature for 12 h. The extracts were filtered, concentrated under reduced pressure and then freeze-dried. Prepared samples were stored at 4 °C until future use.

### 2.2. ABTS and DPPH Radical Scavenging Assay

The ABTS radical solution was generated using 1 mM 2,2’-Azobis(2-amidinopropane) dihydrochloride and 2.5 mM 2,2’-azino-bis (3-ethylbenzothiazoline-6-sulphonic acid) in 100 mL of potassium phosphate buffer solution (pH 7.4). The solution was heated at 70 °C for 30 min and then diluted with EtOH. The test was performed by adding 1470 µL of ABTS radical solution to 30 µL of samples. The mixture was kept at 37 °C for 10 min in the dark before the absorbance at 734 nm was measured.

For DPPH assay, 50 µL of samples with different dilutions was added to 1450 µL of 0.1 mM DPPH in 95% EtOH. The mixture was shaken vigorously and left in the dark for 30 min at room temperature then the absorbance of the mixture was measured at 517 nm using spectrophotometer. The results were expressed as vitamin C equivalents of antioxidant capacity.

### 2.3. Animal Study

Female 7-week-old C57BL/6J mice (Central Lab. Animal Inc., Seoul, Korea) were maintained under controlled conditions of temperature and humidity with 12-h light-dark cycles. All mice were provided with a standard chow diet and water ad libitum. Based on previous reports with acute model of ALD [19], liver damage was induced by three consecutive oral administrations of EtOH at a dose of 6 g/kg and 12 h intervals. The control group received the same volume of isocaloric maltose-dextrin. The water extract powder of *G. bimaculatus* (GBE) and silymarin (positive control) were dissolved in saline, and daily oral administration at 200 mg/kg was initiated 2 weeks before EtOH treatment, along with administration of saline vehicle. At 6 h after the last EtOH dose, the mice were euthanized using an overdose of avertin, and blood was collected by cardiac puncture and centrifuged at 15,000 *g* at 4 °C for 20 min, and then serum was collected. The liver and small intestine were isolated for immunohistochemical staining and molecular analysis. All procedures were carried out in accordance with the institutional guidelines for the use and care of laboratory animals and were approved by the Ethical Committee of the Korea University (Protocol Number: KUIACUC-2018-0035).

### 2.4. Histological Analysis

The isolated liver and small intestine were fixed in 10% neutral buffered formalin, dehydrated using a graded series of alcohol, and embedded in paraffin. The sections (3 μm) were deparaffinized, rehydrated, and stained with hematoxylin and eosin (H&E). For immunofluorescence, the sections were subjected to antigen-retrieval process with 50 mM tris-buffered saline (TBS) and 0.05% Tween-20 in 0.9% saline. Next, the slides were blocked with 1% bovine serum albumin (BSA), 0.3% Triton X-100, and 0.05% sodium azide in phosphate-buffered saline (PBS) for 1 h and incubated overnight at 4 °C with primary antibodies listed in Table S1. Terminal deoxynucleotidyl transferase dUTP nick end labeling (TUNEL) assay was performed by using an in situ cell death detection kit (Roche Diagnostics, Basel, Switzerland), as specified by the manufacturer. Images were acquired using a fluorescence microscope (Carl Zeiss AG, Oberkochen, Germany). 

### 2.5. Western Blot Analysis

Liver and small intestine tissues were homogenized in a lysis buffer containing a phenylmethylsulfonyl fluoride (Roche, Mannheim, Germany) and protease inhibitor cocktail (Sigma, Deisenhofen, Germany) to prepare protein lysates. Protein were separated in a 12% sodium dodecyl sulfate (SDS)-polyacrylamide gel and transferred to nitrocellulose membranes (GE healthcare, Piscataway, NJ, USA). The membranes were blocked with 5% BSA and 0.1% Tween-20 in PBS solution for 1 h at room temperature with constant agitation. The membranes were then probed with the primary antibodies listed in Table S1, followed by incubation with the corresponding horseradish peroxidase-conjugated secondary antibodies (Sigma-Aldrich, St. Louis, MO, USA). The protein bands were visualized using enhanced chemiluminescence reagents on an ImageQuant LAS-4000 imager (General Electric, Pittsburgh, PA, USA) and quantified using the ImageJ software (NIH).

### 2.6. Cell Culture

RAW264.7 cells (a murine macrophage line) were obtained from Korea Research Institute of Bioscience & Biotechnology (KRIBB, Seoul, Korea). Cells were grown in DMEM supplemented with 10% fetal bovine serum (Biowest, Salt Lake City, UT, USA) and 10% penicillin-streptomycin (Gibco, Paisley, Scotland). Cultures were maintained at 37 °C in a 5% CO_2_ humidified atmosphere. When cell treatments were conducted, the cells were incubated in serum-free medium for 6 h, then treated with 200 ng/mL of lipopolysaccharide (LPS) for 6–12 h. For immunoblotting, the cells were washed with PBS, incubated in a lysis buffer at 4 °C for 30 min, and sonicated three times for 10 s. The lysate was centrifuged at 15,000 *g* at 4 °C for 20 min, and the denatured proteins were analyzed.

### 2.7. Biochemical Assays

For hepatic triglyceride (TG) measurement, tissue saponification in ethanolic KOH and neutralization with MgCl_2_ were performed as previously described [20]. Glycerol content was determined by enzymatic colorimetric methods using a commercially available kit (Sigma-Aldrich).

For the study of cell culture, the levels of nitric oxide (NO) in the culture medium were measured using a Griess reagent system kit (Promega, Madison, WI, USA) in accordance with the manufacturer’s protocol. Interleukin-6 (IL-6) and tumor necrosis factor-α (TNF-α) values were measured by an ELISA kit (Invitrogen, Carlsbad, CA, USA) as specified by the manufacturer.

### 2.8. Statistical Analysis

All statistical analyses were performed by one-way ANOVA using SAS software (SAS Institute Inc., Cary, NC, USA). The least squares mean option using Tukey–Kramer adjustment was used for multiple comparisons among the experimental groups. Results were expressed as the means ± SEM. A value of *p* < 0.05 was considered statistically significant.

## 3. Results

### 3.1. GBE Restrains Alcohol-Induced Hepatic Steatosis

Because the use of antioxidants is considered as a rational strategy to prevent liver diseases involving oxidative stress [16], we first tested antioxidant capacities of *G. bimaculatus* extracts prepared with different extracting solvents. ABTS (for water extract) and DPPH (for 70% EtOH extract) assay showed vitamin C equivalent antioxidant capacities of 47.1 and 25.4 mg/g sample, respectively (data not shown). In this study, we used water extract to evaluate protective effects of *G. bimaculatus* against ALD. The crude protein content of water extracts was 66.2% (data not shown).

**Figure 1:**
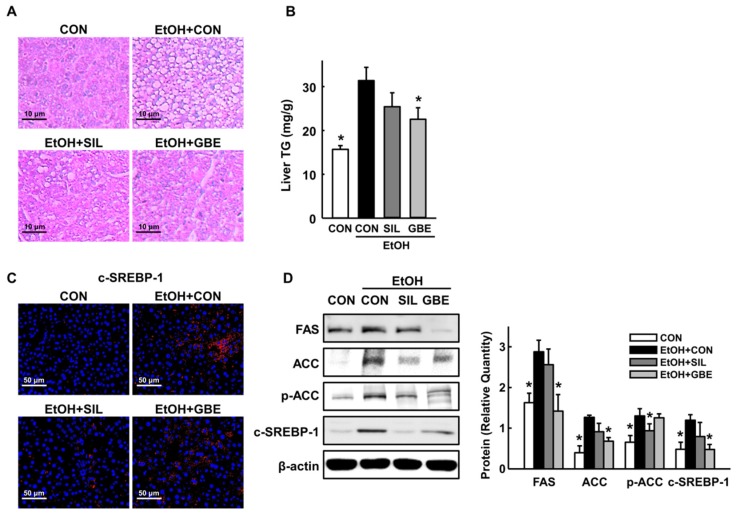
Protective effects of GBE against alcohol-induced hepatic steatosis. (**A**) Representative hematoxylin and eosin (H&E) staining of the liver, (**B**) Liver triglyceride (TG) contents, (**C**) Representative immunohistochemical staining for hepatic cleaved sterol regulatory element-binding protein 1 (SREBP-1) (red), (**D**) Western blot of hepatic fatty acid synthase (FAS), acetyl-CoA carboxylase (ACC), phospho-ACC (p-ACC), and cleaved SREBP-1 expression with quantitative data. CON, control; SIL, silymarin; GBE, water extract of *G. bimaculatus*. Data are shown as means ± SEM (*n* = 4 for A and C and *n* = 8 for B and D). * *p* < 0.05 vs. EtOH control group.

In the present study, three-time administration of EtOH at 12 h intervals induced significant hepatic lipid accumulation (Figure 1A,B), consistent with the observation of increased lipid accumulation in rodent models of acute binge EtOH administration [19]. However, alcohol-induced microvesicular fat infiltration in hepatocytes and hepatic TG accumulation were decreased by GBE administration. To further explore the regulatory effect of GBE on fatty liver, we analyzed key enzymes involved in the de novo synthesis of fatty acids. Control mice exposed to alcohol showed increased hepatic expression levels of fatty acid synthase (FAS), acetyl-CoA carboxylase (ACC), and cleaved sterol regulatory element-binding protein 1 (SREBP-1) that were reduced by GBE (Figure 1C,D). The protective effects of GBE were more potent than those of silymarin at the same dose of treatment. These data indicate that GBE effectively restrains alcohol-induced hepatic steatosis in mice.

### 3.2. GBE Attenuates Alcohol-Induced Hepatic Apoptosis by Suppressing Oxidative Stress

**Figure 2:**
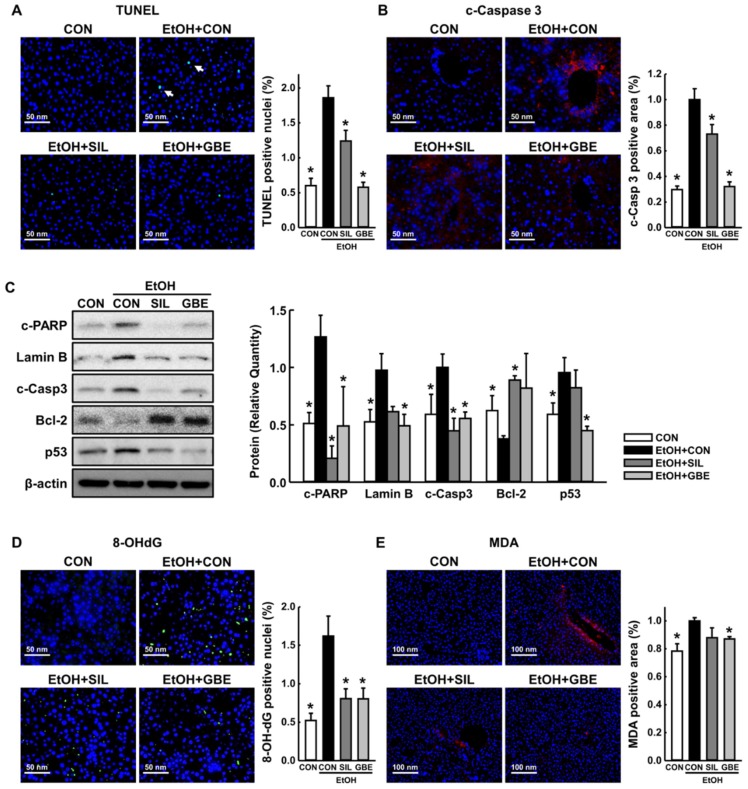
Protective effects of GBE against alcohol-induced hepatic apoptosis and oxidative stress. Representative immunohistochemical staining of liver for (**A**) terminal deoxynucleotidyl transferase dUTP nick end labeling (TUNEL) (green) and (**B**) cleaved caspase-3 (red) with corresponding quantitative data. (**C**) Western blot of hepatic cleaved poly (ADP-ribose) polymerase (PARP), lamin B, cleaved caspase-3, B-cell lymphoma 2 (Bcl-2), and p53 expression with quantitative data. Representative immunohistochemical staining of liver for (**D**) 8-hydroxy-2’-deoxyguanosine (8-OHdG) (green) and (**E**) malondialdehyde (red) with corresponding quantitative data. CON, control; SIL, silymarin; GBE, water extract of *G. bimaculatus*. Data are shown as means ± SEM (*n* = 4 for A, B, D and E and *n* = 8 for C). * *p* < 0.05 vs. EtOH control group.

Progressive hepatocellular failure by apoptosis is central to the pathogenesis of ALD [21]. To assess whether GBE prevents ALD by suppressing alcohol-induced hepatocellular apoptosis, we performed TUNEL assay and examined levels of hallmark proteins of apoptosis in the liver tissue. In EtOH-control mice, we observed a significant increase in hepatic apoptosis as indicated by elevated signals of TUNEL and cleaved caspase-3 (Figure 2A,B). Immunoblotting showed increased protein expressions of cleaved poly (ADP-ribose) polymerase (PARP), lamin B, and cleaved caspase-3 but decreased B-cell lymphoma 2 (Bcl-2) expression in the liver of mice exposed to alcohol (Figure 2C). However, GBE attenuated these apoptotic changes, indicating protective effects of GBE against alcohol-induced hepatic apoptosis. Moreover, tumor suppressor p53, which is increased by DNA damage response and induces transcriptional activation of pro-apoptotic factors [22], was up-regulated by alcohol treatment, but not by alcohol with GBE (Figure 2C).

Since alcohol-induced overproduction of ROS leads to hepatic apoptosis via mechanism of oxidative stress [23], we next measured the biomarkers of oxidative stress in the liver. Immunostaining showed that formations of 8-hydroxy-2’-deoxyguanosine (8-OHdG) and malondialdehyde—representative products of oxidative DNA damage and lipid peroxidation, respectively—were increased by alcohol exposure, but reversed by both silymarin and GBE treatments (Figure 2D,E). However, in this study, we did not observe significant changes in serum activity of alanine aminotransferase among all EtOH-treated groups (data not shown). Taken together, these results indicate that GBE, in part by suppressing ROS-induced oxidative stress, inhibits hepatic apoptosis in mice exposed to alcohol.

### 3.3. GBE Inhibits Kupffer Cell Activation In Vivo and Modulates Inflammatory Response in Macrophages

**Figure 3:**
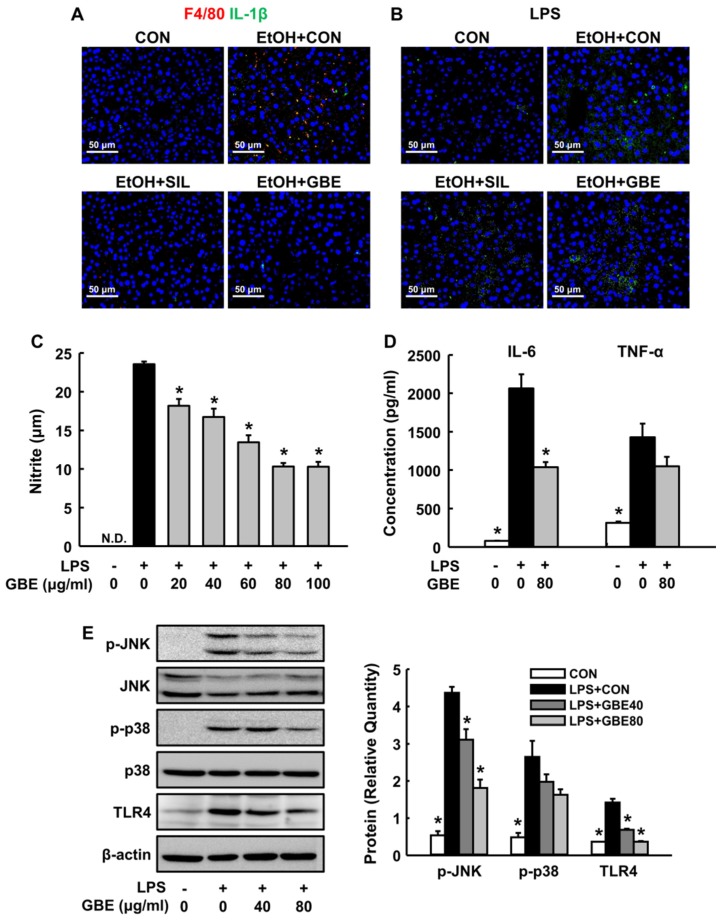
Effects of GBE on alcohol-induced Kupffer cells activation and lipopolysaccharide (LPS)-stimulated inflammatory response in macrophages. Representative immunohistochemical staining of liver for (**A**) F4/80 (red, Kupffer cells marker) and interleukin-1β (green) and (**B**) lipopolysaccharide (green). The released levels of (**C**) nitrite, (**D**) interleukin-6, and tumor necrosis factor-α from lipopolysaccharide-stimulated RAW 264.7 macrophage cells. (**E**) Western blot of phospho-c-Jun N-terminal kinase (p-JNK), total JNK, p-p38, total p38 and toll-like receptor 4 (TLR4) from macrophage cell extracts with quantitative data. CON, control; SIL, silymarin; GBE, water extract of *G. bimaculatus*. Data are shown as means ± SEM (*n* = 4 for A–C and *n* = 6 for D–F). * *p* < 0.05 vs. EtOH control group or LPS control group.

Accumulating evidences have demonstrated that the activation of Kupffer cells (KCs) and related inflammatory cascade play critical roles in the pathogenesis of both chronic and acute ALD [24]. To investigate inflammatory responses in our model, we first observed F4/80-positive signal as a representative marker to monitor KCs in the liver [25]. As shown in Figure 3A, F4/80^+^ KCs and IL-1β were obviously found in the EtOH-control group, which were significantly reduced by both silymarin and GBE treatments. In addition, alcohol treatment induced increased signals of LPS in the liver, but it was attenuated by silymarin and GBE (Figure 3B).

Since hepatic translocation of gut-derived endotoxin acts as an inducer of KC activation through toll-like receptor 4 (TLR4) signaling [24], we next studied LPS-stimulated RAW 264.7 macrophage cells as a surrogate model of KCs activation in ALD [26]. GBE treatment significantly reduced the release of NO and IL-6, which were increased by LPS stimulation in macrophages (Figure 3C,D). Moreover, GBE reduced LPS-induced increase in TLR4 expression and phosphorylation of JNK and p38 (Figure 3E), suggesting that GBE inhibits KC activation by suppressing hepatic translocation of endotoxin and TLR4-mediated mitogen-activated protein kinase (MAPK) signaling.

### 3.4. GBE Protects the Intestine against Alcohol-Induced Hyperpermeability and Oxidative Stress

**Figure 4:**
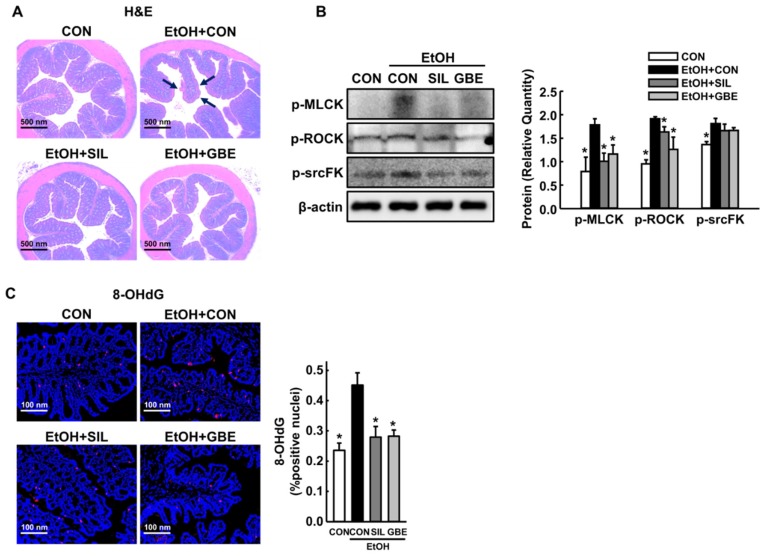
Protective effects of GBE against alcohol-induced intestinal hyperpermeability and oxidative stress. (**A**) Representative H&E staining of the small intestine, (**B**) Western blot of intestinal phospho-myosin light chain kinase (p-MLCK), phospho-Rho-associated protein kinase (p-ROCK), and phospho-Src-family kinase (p-srcFK) with quantitative data, (**C**) Representative immunohistochemical staining for intestinal 8-OHdG with quantitative data. CON, control; SIL, silymarin; GBE, water extract of *G. bimaculatus*. Data are shown as means ± SEM (*n* = 4 for A and C and *n* = 8 for B). * *p* < 0.05 vs. EtOH control group.

Alcohol exposure increases intestinal permeability to bacterial endotoxin by promoting phosphorylations of tight and adherens junction proteins, resulting in increased hepatic inflammation via KCs activation [27]. Because GBE suppressed hepatic translocation of LPS in this study (Figure 3B), we next examined effects of GBE on alcohol-induced intestinal damage. EtOH exposure caused significant injury to the small intestine, as shown by loss of epithelial cells of the villi and submucosal blebbing (Figure 4A). In contrast, GBE- or silymarin-administered mice showed no observable alterations in the small intestine after EtOH treatment. Importantly, GBE attenuated EtOH-induced intestinal phosphorylation of myosin light chain kinase, Rho-associated protein kinase, and Src-family kinase (Figure 4B), which are major regulators of tight and adherent junctions [28,29]. Consistent with the reduction of hepatic oxidative stress (Figure 2), GBE also reduced intestinal 8-OHdG increased by alcohol exposure (Figure 4C). Thus, these findings indicate that GBE counteracts oxidative stress-induced tight junction remodeling and hyperpermeability in the small intestine, thereby protecting against alcohol-stimulated intestinal damage in mice.

## 4. Discussion

**Figure 5:**
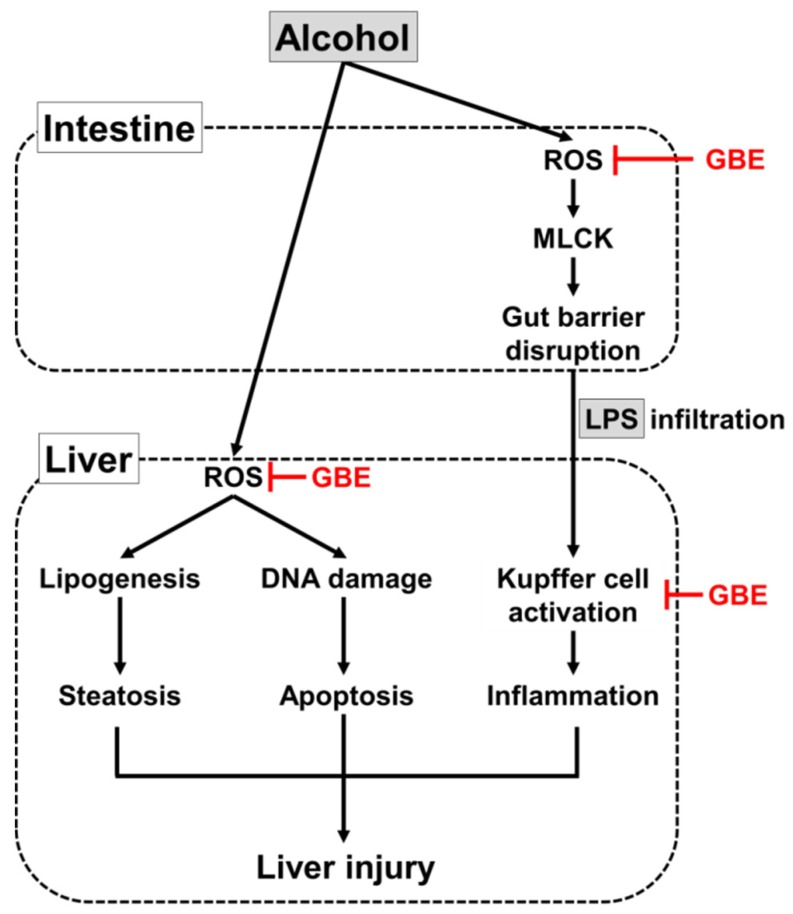
Proposed mechanism of protection of GBE against alcohol-induced liver injury.

With growing interest in the use of edible insects as a functional food resource, here we demonstrated that GBE counteracts acute ALD in mice via suppression of EtOH-associated oxidative stress and inflammatory responses in the liver and small intestine. Figure 5 shows proposed action mechanisms of GBE. The first finding of this study was that GBE attenuated alcohol-induced hepatic steatosis and apoptosis. Its effects were comparable to or more potent than silymarin, a known therapeutic agent for ALD [30]. Of note, these protective effects of GBE were associated with inhibition of ROS-induced oxidative damage. Ethanol and its metabolites induce the overproduction of ROS in liver cells, and ROS directly affects the transcriptional network that controls lipid metabolism and apoptotic response during liver injury [14,31]. Along with the significance of ROS and oxidative stress in the pathogenesis of ALD, a number of studies have demonstrated that natural antioxidants have preventive effects on ALD in different model systems [16]. In line with previous evidence supporting the use of antioxidants as a rational strategy to prevent and/or treat ALD, our current data suggest the use of edible insect *Gryllus bimaculatus* as an effective option.

Alcohol-induced reactive oxygen species (ROS) and oxidative stress induce hepatic steatosis and apoptosis through lipogenic pathway and DNA damage response, respectively. In addition, ROS promote gut barrier disruption and subsequent LPS infiltration into the liver, resulting in inflammatory responses via Kupffer cells activation during alcoholic liver injury. GBE may protect the liver from alcoholic damage by suppressing ROS-induced oxidative stress in both intestine and liver and Kupffer cell activation. Black and red lines indicate stimulatory and inhibitory actions, respectively.

The second observation was that GBE protected against alcohol-induced intestinal barrier dysfunction and subsequent KC activation. Accumulating evidence suggests that gut-derived bacterial endotoxins (LPS) play a critical role in the development of ALD [27,32]. Alcohol and its metabolites disrupt intestinal epithelial barrier by altering expression and/or phosphorylation of tight junction proteins, leading to increased release of endotoxin into portal circulation. Afterward, excess amounts of endotoxin reaching the liver act as an inflammatory signal that activates KCs through TLR4-mediated pathways [27,33]. Therefore, preserving intestinal integrity and suppressing the transfer of endotoxin in the gut–liver axis have been suggested as logical strategies for the protection of liver from alcoholic injury [32]. In this study, we observed that GBE reversed alterations of tight and adherens junction proteins in the intestine by alcohol and consequently blocked LPS-stimulated inflammatory responses in the liver. Moreover, GBE suppressed intestinal oxidative stress as shown by reduced 8-OHdG formations, and this may be an important initial point to explain the hepatoprotective effects of GBE. Indeed, it is well-established that excess ROS generated from alcohol metabolism and oxidative stress in the intestine promote changes in tight junction proteins and related cellular signaling, thereby contributing to development of gut leakiness in ALD [33,34]. Taken together, our data suggest that GBE effectively counteracts alcohol-induced hepatic failures in mice, in part by suppressing ROS-induced oxidative stress in both small intestine and liver tissues.

It has been previously reported that glycosaminoglycans derived from *G. bimaculatus* exert anti-inflammatory and anti-lipidemic properties in rodent models [8,9], although their underlying mechanisms remain unclear. In addition, in carbon tetrachloride-induced liver fibrogenic rat models, glycosaminoglycans hyaluronic acid and chondroitin-4-sulphate restored hepatic injury, concomitant with enhanced antioxidant enzyme activity in the liver [35]. These reports suggest the role of glycosaminoglycans as one of possible bioactive compounds in *G. bimaculatus*. Indeed, glycosaminoglycans are main components of the hepatic extracellular matrix, and several researches have suggested the important roles of glycosaminoglycans in liver regeneration, along with observations of significant increases in their physiological levels during the progression of liver injury [36,37]. In the GBE used in this study, chondroitin-4-sulphate content was 2.38%, but hyaluronic acid was not detected (data not shown). However, further studies are needed to investigate bioactive compounds responsible for the protective effects of GBE in ALD.

In summary, this study showed that GBE administration ameliorated acute alcohol-induced liver injury in mice via inhibition of hepatic steatosis, apoptosis, and gut-derived inflammatory responses, possibly by suppressing oxidative stress. These findings provide mechanistic insight into the protective effects of GBE against ALD, and further studies are needed to investigate the long-term effects of GBE in chronic ALD model for its utilization as a promising hepatoprotective agent. The animal model used in this study is limited to alcohol-related damage and may not reflect other types of hepatotoxicity.

## Supplementary Materials

The following are available online at https://www.mdpi.com/2072-6643/11/4/857/s1, Table S1: Antibodies used for western blotting and immunofluorescent staining.

## References

[1] Van Huis A., Van Itterbeeck J., Klunder H., Mertens E., Halloran A., Muir G., Vantomme P. (2013). Edible insects: Future Prospects for Food and Feed Security.

[2] Belluco S., Losasso C., Maggioletti M., Alonzi C.C., Paoletti M.G., Ricci A. (2013). Edible insects in a food safety and nutritional perspective: A critical review. Compr. Rev. Food Sci. Food Saf..

[3] Dobermann D., Swift J.A., Field L.M. (2017). Opportunities and hurdles of edible insects for food and feed. Nutr. Bull..

[4] Ahn M.Y., Hwang J.S., Yun E.Y., Kim M.-J., Park K.-K. (2015). Anti-aging effect and gene expression profiling of aged rats treated with *G. bimaculatus* extract. Toxicol. Res..

[5] Ahn M.Y., Kim M.-J., Kwon R.H., Hwang J.S., Park K.-K. (2015). Gene expression profiling and inhibition of adipose tissue accumulation of *G. bimaculatus* extract in rats on high fat diet. Lipids Health Dis..

[6] Dong-Hwan S., Hwang S.Y., Han J., Koh S.K., Kim I., Ryu K.S., Yun C.Y. (2004). Immune-Enhancing Activity Screening on Extracts from Two Crickets, *Gryllus bimaculatus* and *Teleogryllus emma*. Entomol. Res..

[7] Im A.-R., Yang W.-K., Park Y.-C., Kim S.H., Chae S. (2018). Hepatoprotective Effects of Insect Extracts in an Animal Model of Nonalcoholic Fatty Liver Disease. Nutrients.

[8] Ahn M.Y., Han J.W., Hwang J.S., Yun E.Y., Lee B.M. (2014). Anti-inflammatory effect of glycosaminoglycan derived from *Gryllus bimaculatus* (a type of cricket, insect) on adjuvant-treated chronic arthritis rat model. J. Toxicol. Environ. Health Part A.

[9] Ahn M.Y., Hwang J.S., Kim M.-J., Park K.-K. (2016). Antilipidemic effects and gene expression profiling of the glycosaminoglycans from cricket in rats on a high fat diet. Arch. Pharmacal Res..

[10] Marcellin P., Kutala B.K. (2018). Liver diseases: A major, neglected global public health problem requiring urgent actions and large-scale screening. Liver Int..

[11] Sakhuja P. (2014). Pathology of alcoholic liver disease, can it be differentiated from nonalcoholic steatohepatitis?. World J. Gastroenterol. WJG.

[12] Ambade A., Mandrekar P. (2012). Oxidative stress and inflammation: Essential partners in alcoholic liver disease. Int. J. Hepatol..

[13] Louvet A., Mathurin P. (2015). Alcoholic liver disease: Mechanisms of injury and targeted treatment. Nat. Rev. Gastroenterol. Hepatol..

[14] Ceni E., Mello T., Galli A. (2014). Pathogenesis of alcoholic liver disease: Role of oxidative metabolism. World J. Gastroenterol. WJG.

[15] Han K.-H., Hashimoto N., Fukushima M. (2016). Relationships among alcoholic liver disease, antioxidants, and antioxidant enzymes. World J. Gastroenterol..

[16] Li S., Tan H.-Y., Wang N., Zhang Z.-J., Lao L., Wong C.-W., Feng Y. (2015). The role of oxidative stress and antioxidants in liver diseases. Int. J. Mol. Sci..

[17] Becker U., Deis A., Sørensen T.I., Grønbaek M., Borch-Johnsen K., Müller C.F., Schnohr P., Jensen G. (1996). Prediction of risk of liver disease by alcohol intake, sex, and age: A prospective population study. Hepatology.

[18] Sato N., Lindros K.O., Baraona E., Ikejima K., Mezey E., Järveläinen H.A., Ramchandani V.A. (2001). Sex difference in alcohol-related organ injury. Alcohol. Clin. Exp. Res..

[19] Ghosh Dastidar S., Warner J.B., Warner D.R., McClain C.J., Kirpich I.A. (2018). Rodent Models of Alcoholic Liver Disease: Role of Binge Ethanol Administration. Biomolecules.

[20] Norris A.W., Chen L., Fisher S.J., Szanto I., Ristow M., Jozsi A.C., Hirshman M.F., Rosen E.D., Goodyear L.J., Gonzalez F.J. (2003). Muscle-specific PPARγ-deficient mice develop increased adiposity and insulin resistance but respond to thiazolidinediones. J. Clin. Investig..

[21] Luedde T., Kaplowitz N., Schwabe R.F. (2014). Cell death and cell death responses in liver disease: Mechanisms and clinical relevance. Gastroenterology.

[22] Haupt S., Berger M., Goldberg Z., Haupt Y. (2003). Apoptosis-the p53 network. J. Cell Sci..

[23] Wang K. (2015). Molecular mechanisms of hepatic apoptosis. Cell Death Dis..

[24] Zeng T., Zhang C.-L., Xiao M., Yang R., Xie K.-Q. (2016). Critical roles of Kupffer cells in the pathogenesis of alcoholic liver disease: From basic science to clinical trials. Front. Immunol..

[25] Tsai T.H., Tam K., Chen S.F., Liou J.Y., Tsai Y.C., Lee Y.M., Huang T.Y., Shyue S.K. (2018). Deletion of caveolin-1 attenuates LPS/GalN-induced acute liver injury in mice. J. Cell. Mol. Med..

[26] Bala S., Marcos M., Kodys K., Csak T., Catalano D., Mandrekar P., Szabo G. (2011). Up-regulation of microRNA-155 in macrophages contributes to increased tumor necrosis factor α (TNFα) production via increased mRNA half-life in alcoholic liver disease. J. Biol. Chem..

[27] Purohit V., Bode J.C., Bode C., Brenner D.A., Choudhry M.A., Hamilton F., Kang Y.J., Keshavarzian A., Rao R., Sartor R.B. (2008). Alcohol, intestinal bacterial growth, intestinal permeability to endotoxin, and medical consequences: Summary of a symposium. Alcohol.

[28] Shen L., Black E.D., Witkowski E.D., Lencer W.I., Guerriero V., Schneeberger E.E., Turner J.R. (2006). Myosin light chain phosphorylation regulates barrier function by remodeling tight junction structure. J. Cell Sci..

[29] MacKay C.E., Shaifta Y., Snetkov V.V., Francois A.A., Ward J.P., Knock G.A. (2017). ROS-dependent activation of RhoA/Rho-kinase in pulmonary artery: Role of Src-family kinases and ARHGEF1. Free Radic. Biol. Med..

[30] Saller R., Meier R., Brignoli R. (2001). The use of silymarin in the treatment of liver diseases. Drugs.

[31] McVicker B.L., Tuma D.J., Casey C.A. (2007). Effect of ethanol on pro-apoptotic mechanisms in polarized hepatic cells. World J. Gastroenterol. WJG.

[32] Szabo G. (2015). Gut–liver axis in alcoholic liver disease. Gastroenterology.

[33] Forsyth C.B., Voigt R.M., Keshavarzian A. (2014). Intestinal CYP2E1: A mediator of alcohol-induced gut leakiness. Redox Biol..

[34] Keshavarzian A., Farhadi A., Forsyth C.B., Rangan J., Jakate S., Shaikh M., Banan A., Fields J.Z. (2009). Evidence that chronic alcohol exposure promotes intestinal oxidative stress, intestinal hyperpermeability and endotoxemia prior to development of alcoholic steatohepatitis in rats. J. Hepatol..

[35] Campo G.M., Avenoso A., Campo S., D’Ascola A., Ferlazzo A.M., Calatroni A. (2004). The antioxidant and antifibrogenic effects of the glycosaminoglycans hyaluronic acid and chondroitin-4-sulphate in a subchronic rat model of carbon tetrachloride-induced liver fibrogenesis. Chem. Biol. Interact..

[36] Bramley P., Rathbone B., Forbes M., Cooper E., Losowsky M. (1991). Serum hyaluronate as a marker of hepatic derangement in acute liver damage. J. Hepatol..

[37] Nadanaka S., Kitagawa H. (2014). EXTL2 controls liver regeneration and aortic calcification through xylose kinase-dependent regulation of glycosaminoglycan biosynthesis. Matrix Biol..

