# Supplementary material for: The Edible Insect Gryllus bimaculatus Protects against Gut-Derived Inflammatory Responses and Liver Damage in Mice after Acute Alcohol Exposure
Source: Nutrients. 2019 Apr 16;11(4):857. doi: 10.3390/nu11040857 (PMC6521266; doi:10.3390/nu11040857)
Supplement: Supplementary file 1 [file nutrients-11-00857-s001.pdf]

Supporting Information

Table S1. Antibodies used for western blotting and immunofluorescent staining.

Primary antibody	Clone	Company	Catalog No.	Dilution
**FAS**	Polyclonal	Thermo	Pa1-32355	1:1000 (WB)
**ACC**	Polyclonal	Thermo	Pa5-17564	1:1000 (WB)
**SREBP-1**	Polyclonal	Thermo	Pa1-46142	1:500 (WB)<br>1:200 (IF)
**c-Caspase3**	Polyclonal	Cell signaling	#9662	1:1000 (WB)<br>1:300 (IF)
**c-PARP**	E51	ABcam	ab32064	1:2000 (WB)
**Lamin B**	C-20	Santa Cruz	Sc-6216	1:2000 (WB)
**Bcl-2**	100/D5	ABcam	ab692	1:500 (WB)
**p53**	Pab 1801	Santa Cruz	Sc-98	1:1000 (WB)
**8-OH-dG**	15A3	ABcam	Ab62623	1:200 (IF)
**MDA**	11E3	NOVUS	NBP2-59367	1:50 (IF)
**IL-1β**	Polyclonal	ABcam	Ab9722	1:50 (IF)
**F4/80**	C-7	Santa Cruz	Sc-377009	1:300 (IF)
**LPS**	WN1 222-5	Hycult Biotech	HM6011	1:100 (IF)
**p-JNK**	EPR5693	ABcam	Ab124956	1:1000 (WB)
**JNK**	EPR16797-211	ABcam	Ab179461	1:1000 (WB)
**p-p38**	Polyclonal	ABcam	Ab47363	1:1000 (WB)
**p38**	M138	ABcam	Ab31828	1:1000 (WB)
**TLR4**	Polyclonal	ABcam	Ab13867	1:1000 (WB)
**p-MLCK**	Polyclonal	ABcam	Ab200809	1:1000 (WB)
**p-ROCK**	Polyclonal	ABcam	Ab203273	1:1000 (WB)
**p-srcFK**	D49G4	Cell signaling	#6943	1:1000 (WB)
**β-actin**	C4	Santa Cruz	Sc-47778	1:2000 (WB)
